# Adherent Placenta, Profuse Hæmorrhage, Compression of the Aorta—Removed—Recovery, &c., &c.

**Published:** 1855-07

**Authors:** Ira E. Oatman

**Affiliations:** Sacramento City, California


ARTICLE III.—*Adherent Placenta, Profuse Hæmorrhage, Compression of the Aorta—Removed—Recovery, &c., &c.* BY IRA E. OATMAN, M. D., of Sacramento City, California.

ON the 20th of March, 1855, I was called in this city to consult with a Doctor and a Doctress—the latter having the case in charge.

Mrs. R. æt. 30, of replete habit, has had some chronic disease of the vagina and perhaps cervix uteri, since the recession of a scrofulous eruption on the skin, some eight years ago. Although married many years, she never conceived until she arrived in California.

After a natural labor, she was delivered of a child at 5 o'clock A. M. The Placenta not coming away, there was considerable hœmorrage until 2 o'clock P. M., when it became profuse. At 3 P. M. the *Doctress* being alarmed, sent for the other counsel, who had me called at 4. I found the patient deadly pale, with livid lips, extremities cold and nails blue. She was "*so tired*," thirsty and vomiting, with syncope upon the slightest exertion. The hœmorrhage still continuing.

I urged the immediate removal of the placenta, as the only immunity from speedy death.

Consent being obtained, and the Doc*tress* and Doc*tor*, both declining the operation, I proceeded as follows: Placing the thumb as a safety valve upon the aorta, with the left hand well lubricated, I succeeded in entering the uterus, and in detaching and removing the placenta with the coagula. The uterus contracting but little, with the aorta still firmly held, I compressed the abdomen tightly with a thin bandage and covered it with ice. In thirty minutes the compression of the aorta was removed and no hœmorrhage followed. The case was safe!

The next day there was considerable reaction, with tenderness of the uterus and of the abdomen where the compression was made. She is having a slow but a safe recovery. Contingencies: 1st. It was difficult to compress the aorta on account of the corpulence of the patient; and, 2d. Eleven and a half hours having elapsed since the delivery of the child, the vulva was so irritable, that efforts to pass the hand produced syncope repeatedly,—so that it required gentle perseverance for fifteen or twenty minutes, to ensure success with safety.

I submit this case to show—1st. That the placenta can be safely detached and removed with the coagula, by using compression of the aorta; when the hæmorrhage incident to that operation without it would be fatal: and 2d. To show the insecurity of ladies during parturition in the case of women, who, though they may have the knowledge, have not the nerve to discharge the responsible duties of the accoucheur under all circumstances;—for, it is obvious in this case, that the placenta should have been detached and removed within an hour or two after delivery—before the hæmorrhage had jeopardized the life of an amiable wife and mother.

Sacramento City, Cal., April 16, 1855.

